# Muriform Cells Can Reproduce by Dividing in an Athymic Murine Model of Chromoblastomycosis due to *Fonsecaea pedrosoi*

**DOI:** 10.4269/ajtmh.19-0465

**Published:** 2020-06-08

**Authors:** Bilin Dong, Wei Liu, Ruoyu Li, Yao Chen, Zhongsheng Tong, Xu Zhang, Liuqing Chen, Dongsheng Li

**Affiliations:** 1 Department of Dermatology, Center for Infectious Skin Diseases, No.1 Hospital of Wuhan, Wuhan, China;; 2 Institute of Applied Mycology, Huazhong Agricultural University, Wuhan, China;; 3 Department of Dermatology, Peking University First Hospital, and Research Center for Medical Mycology, Peking University, Beijing, China

## Abstract

Transformation of *Fonsecaea pedrosoi* into muriform cells enhances the resistance against phagocytosis and elimination by host immune cells, and links to the chronicity of chromoblastomycosis. Here, we aim to determine whether the muriform cells can reproduce in tissue without reverse transformation into hyphal form by using an experimental nu/nu-BALB/c mouse model of chromoblastomycosis due to *F. pedrosoi*. During the whole 81-day observation period, most of the hyphal inocula had transformed into muriform cells at 75 days postinoculation and maintained as this parasitic morphology till 81 days postinoculation simultaneously with increased fungal loads in tissue and the worsening of footpad lesion. Scanning and transmitting electronic microscope examinations showed that the muriform cells obtained in tissue or induced in vitro can reproduce daughter cells by dividing, and, meanwhile, the daughter cells had the potential to produce buds and grow into hyphae reversely. Furthermore, exoenzyme examination suggested that the profile of exoenzymes constituted by muriform cells was quite different from that constituted by hyphae although the assay showed both of them had obvious metabolic activity. By contrast, most muriform cells in the footpad gradually transformed into the elongated hyphae without obvious infiltration of inflammatory cells during repeated intraperitoneal administration of cyclophosphamide (50 mg/kg, per every other day) from 50 to 80 days postinoculation. Therefore, we infer that *F. pedrosoi* can reproduce by dividing as muriform cells in mouse tissue, and the morphological transformation between hyphal form and muriform cells is possibly associated with the host immune status.

## INTRODUCTION

Chromoblastomycosis is a chronic granulomatous mycosis of the skin and subcutaneous tissues caused by melanized fungi, of which *Fonsecaea pedrosoi* is considered as one of the most common agents.^1–3^ This disease prevails in tropical or subtropical zones worldwide and usually affects the outdoor laborers living in low-income regions of Asia, Africa, and Latin America.^3^ Although the lesions of chromoblastomycosis progress slowly and limit to the subcutaneous tissues, this disease gradually produces fibrotic changes and lymphatic stasis with clinical complications including lymphedema and malignant transformation of such long-standing lesions.^3–6^ The effects of oral antifungal therapies for this disease leave much to be improved.^1–3,7^ Currently, the chromoblastomycosis has been included in neglected tropical diseases by the WHO.^3–6^

Characteristically, when embedded in tissue, most etiological agents of this disease including *F. pedrosoi* will transform into the parasitic form, that is, the muriform cells with transverse and longitudinal cross-walls.^3,7,8^ Some data showed that this morphological change contributes to the resistance against host immune response and, therefore, drives the chronicity of this disease.^9–12^

Of note, the budding from muriform cells and hyphal extension were observed in several cases of extensive chromoblastomycosis.^13,14^ What is more, invasive hyphal growth without transformation into muriform cells was observed in some special cases of corneal infection caused by *F. pedrosoi*.^15–17^ These phenomena cohere with the term “sclerotic cells,” which was derived from “sclerotia,” and explained the parasitic form of chromoblastomycosis agents as compacted masses of latent hyphae.^3^

However, some other studies suggested that the parasitic form of *F. pedrosoi* can present a thick multilayer and electron-dense cell wall with transverse septum that is peculiar to planate division and meristematic growth, from which the name “muriform cells” was derived.^3,18^

Considering that the transformation into muriform cells can enhance the ability of parasitic *F. pedrosoi* to defend against host elimination and is involved in some immune escape mechanisms as mentioned earlier,^3,11,12^ it is essential to investigate whether the muriform cells can divide to form daughter cells in the infected tissue without reverse transformation into the hyphal form. But in reality, it is difficult to actualize dynamic monitoring for the reproduction mode of chromoblastomycosis agents in tissue at the patient’s level.

Although the immunocompetent BALB/c mice inoculated subcutaneously with *F. pedrosoi* developed to be self-healing, athymic (nu/nu−) BALB/c mice inoculated subcutaneously with the agent were prone to be chronically infected, and, thus, partly reflect the clinical and histopathological characteristics of human chromoblastomycosis.^19–23^

In the present study, we used an athymic murine model of chromoblastomycosis due to *F. pedrosoi* introduced by previous studies with an aim to determine whether the muriform cells have self-reproducing activity without reverse transformation into the hyphal form in tissue by shortening the observation period to a controlled range.^20–23^ In addition, we further analyzed whether there existed possible linkage between the parasitic form of *F. pedrosoi* in tissue and host immune status by intraperitoneal administration of cyclophosphamide (CTX).

## MATERIALS AND METHODS

### Source of mice.

Athymic (nu/nu) BALB/c male mice (special pathogen free [SPF]; 5–6 weeks old) were purchased from the Animal Laboratory Center, Wuhan University, and maintained in special pathogen-free conditions.

### Compliance with ethical standards.

We adhered to the guidelines stated in the Belmont Report and those set forth by the Council for International organizations of Medical Sciences in experimental use of animals. All procedures performed in the present study involving the animals (nu/nu-BALB/c mice) complied with the guidelines for humane use of laboratory animals from the National Institute of Health and were in accordance with the ethical standards of the institution at which the study was conducted (the Institutional Animal Care and Use Committee of No.1 Hospital of Wuhan, project permit number: WHB201511012).

### Fungal strain and preparation of hyphal fragments.

The fungal strain (WH10-002) was isolated from the skin lesion of the chromoblastomycosis patient and was identified as *F. pedrosoi* by DNA sequencing at “ITS1+5,8SrRNA+ITS2” region (GenBank number: GQ420654.1). The strain was cultivated on potato dextrose agar (PDA) (Ref 213400, Difco^™^, BD, Sparks, MD) supplemented with chloramphenicol at 50 μg/mL at 28°C and was periodically transferred at 60-day intervals for preservation.

To prepare *F. pedrosoi* hyphae, the stock culture was inoculated into the Sabouraud dextrose broth (SDB) (Ref 238220, Difco^™^ BD, Sparks, MD) and cultured for 2 weeks at 28°C. Afterward, the mycelial masses in broth were unfolded with a glass homogenizer by pushing and pulling the plunger gently for several times, and then the homogenous suspension was filtered through a nylon filter (200 mesh), where the remnant mycelium masses were retained. The filtrate containing solitary, short hyphal fragments was further washed twice in normal saline (NS) by centrifugation at 4,000 rpm for 5 minutes for use. The viability rate of hyphal fragments was detected by FUN1 cell stain (Cat: F7030, Invitrogen^™^, Eugene, OR) using a flow cytometer according to the protocol and reached greater than 96%.

To determine the concentration of hyphal inocula, the original filtrate was diluted by adding the sterile normal saline according to the ratio of 1:10, 1:100, 1:250, 1:500, 1:1,000, 1:2,500, 1:5,000, and 1:10,000. Afterward, the turbidity of each diluent was, respectively, measured by DensiCHEK plus (BioMericux, Durham, NC). Simultaneously, 100 μL of each diluent within the proper readable range (1:250–1:5, 000) was further coated onto the PDA plate (*n* = 5 for each indicated dilution) at 28°C for 5 days, and the colonies growing on the plate were counted and represented as colony-forming unit (CFU). According to the linear regression curve established by CFU and turbidity, the hyphal inocula was adjusted to a final concentration of 1.5 × 10^8^ CFU/mL before inoculation.

### Footpad infection with *F. pedrosoi* hyphae.

Ten minutes before infection, the nu/nu-BALB/c mice were anesthetized by intraperitoneal injection with 0.4 μL of Anasedan and 0.35 mL of Dopalen per kg body weight. Afterward, 100 μL of *F. pedrosoi* hyphae inocula with a concentration of 1.5 × 10^8^ CFU/mL was injected subcutaneously into each rear footpad. The mice injected subcutaneously with 100 μL of normal saline were set as inoculation control.

### Monitoring of fungal morphology in tissue.

A morphological change of the inoculated *F. pedrosoi* hyphae in the purulent discharge obtained from the same infected footpad was consecutively monitored at 7, 40, 75, and 81 days postinoculation. In addition, tissue biopsy of the infected footpads and hematoxylin-eosin (HE) staining were performed at 81 days postinoculation to further determine whether most of the inoculated *F. pedrosoi* hyphae had transformed into muriform cells in tissue.

### Evaluation of footpad lesion and fungal burden.

The development of footpad lesion was monitored twice a week for up to a maximum of 81 days, and footpad swelling was simultaneously measured using a vernier caliper. Footpad volume was calculated by length × width × thickness measurements, and the data were represented as mean ± standard error of the mean for the infected group as well as the inoculation control (*n* = 5 for each group). The images for the same infected footpad were taken consecutively at 4, 7, 12, 24, 40, 75, and 81 days postinoculation, and the pre-inoculation image was taken at 0 day.

For evaluation of fungal burden, the mice subcutaneously inoculated with *F. pedrosoi* hyphae were sacrificed, and the infected footpads were aseptically isolated, respectively, at 75 and 81 days postinoculation (*n* = 4 at each indicated time point). Then, the mouse footpads were homogenized with glass tissue homogenizers, and the homogenous suspension was filtered through a nylon filter (200 mesh). A total of 2 mL of filtrate from each sample was collected and 10-fold serially diluted (1–10^3^) in sterile normal saline with penicillin (500 U/mL) and streptomycin (500 μg/mL). Afterward, 100 μL of the original filtrate or each diluent was coated onto each PDA plate containing 50 mg/L of chloramphenicol (*n* = 5 for each indicated dilution) at 28°C for 5 days. The fungal load in the infected footpad was measured by counting fungal colonies on the plate inoculated with appropriate diluents and was represented as the CFU.

### Administration of CTX after hyphal inocula of *F. pedrosoi* transformed into muriform cells.

Another group of nu/nu-BALB/c mice (*n* = 5) were inoculated subcutaneously with *F. pedrosoi* hyphal fragments (1.5 × 10^8^ CFU/mL, 100 μL per rear footpad) as described earlier. A morphological change of hyphal inocula in the purulent discharge obtained from the same infected footpad was consecutively monitored until most hyphal inocula had transformed into the muriform cells at 50 days postinoculation. Each mouse was then given intraperitoneally 300 μL of CTX at a dose of 50 mg/kg every other day from 50 to 80 days postinoculation. In this period, the morphology of the agent in the purulent was simultaneously monitored. The development of footpad lesion was also monitored during the whole 80-day observation period, and the images were taken at 3, 7, 15, 21, 30, 50, 60, 70, and 80 days postinoculation, respectively. In addition, tissue biopsy of the infected footpads and HE staining were performed at 80 days postinoculation to further determine the morphology of *F. pedrosoi* in tissue after CTX administration.

### In vitro induction of muriform cells.

To induce the formation of muriform cells, the 15-day old *F. pedrosoi* hyphae grown in SDB were unfolded with a glass homogenizer and adjusted to a final concentration of 0.5 × 10^6^ CFU/mL as mentioned in the “Fungal strain and preparation of hyphal fragments” section. Then, 500 μL of hyphal fragments was reinoculated into 30 mL of synthetic basal medium (ATCC medium 830), pH 5.5, with the following composition (g/L): MgSO_4_, 0.1; NH_4_NO_3_, 1.5; KH_2_PO_4_, 1.8; biotin, 5 × 10^−5^; thiamine-HCl, 1.0 × 10^−4^; and glycerol, 6.5, as previously described.^8,11,20,24^ In addition, Nikkomycin Z (N8028, Sigma-Aldrich, St. Louis, MO) was added at a final concentration of 50 μg/mL. During the 50-day incubation period at 35°C, the formation of muriform cells was confirmed by microscopic examination.

### Ultrastructural examination by scanning and transmitting electronic microscopes (SEM and TEM).

In vitro–induced muriform cells after incubation for 50 days and the infected footpad tissues with or without CTX administration, respectively, at 80 or 81 days postinoculation were collected and fixated with 2.5% glutaraldehyde solution at 4°C for more than 24 hours. Afterward, the specimens were sent to the Wuhan Institute of Virology and Institute of Hydrobiology, Chinese Academy of Science for SEM (SU-8010/S-4800, Hitachi, Japan) and TEM (Tecnai G^2^ 20 TWIN, FEI, Hillsboro, OR/HT 7700, Hitachi, Japan) examinations.

### Assay for metabolic activities.

To determine the metabolic activities of muriform cells obtained in vitro and in vivo as well as saprophytic hyphae, the FUN-1 cell stain (Cat: F7030, Invitrogen^™^, Eugene, OR) was used. According to the protocol, metabolically active fungal cells can process the intracellular FUN-1 dye and produce orange–red fluorescent intra-vacuolar structures, whereas cells with little or no metabolic activities exhibit original green cytoplasmic fluorescence.

Briefly, 300 μL of *F. pedrosoi* hyphae or muriform cell suspension was prepared, respectively, and adjusted to 5.0 × 107 CFU/mL in PBS solution. Subsequently, 3 μL of FUN-1 stock solution (10 mmol·L^−1^) was added in each of them, and the suspensions were incubated at 37°C for 1 hour with shaking at 50 rpm. Afterward, the fluorescence distribution of FUN-1 dye in fungal cells was measured by using confocal microscope (Leica TCS SP8, Germany). As suggested in the protocol, the excitation wavelength was set as 488 nm, the green fluorescence emission was detected at 510–550 nm wavelength range, and the red fluorescence emission was detected at the 570–610 nm wavelength range.

### Assay for exoenzyme profiles.

To determine the exoenzyme profiles constituted by *F. pedrosoi* hyphae, muriform cells in vivo and in vitro, the API ZYM strip (Ref 25200, BioMericux, France) was used in the present study. As the protocol suggested, this strip is applicable to microorganisms including fungi and allows the systematic and rapid study of 19 exoenzymatic reactions.

Briefly, the suspensions of *F. pedrosoi* hyphae, muriforms in vitro and in vivo were, respectively, prepared and adjusted to 2.0 × 108 CFU/mL in 2 mL of API Suspension Medium (Ref 70700, BioMericux, France). Subsequently, 65 μL of fungal specimen was dispensed into each cupule of the strip. After incubation for 5 hours at 37°C, ZYM A (Ref 70494, BioMericux, France) and ZYM B (Ref 70493, BioMericux, France) reagents were, respectively, added into each cupule according to the protocol. The criteria for the positive or negative reactions are mentioned in Table 1.

**Table 1:** Exoenzymes produced by *Fonsecaea pedrosoi* hyphae and muriform cells in vivo and in vitro

No.	Exoenzyme to be assayed*	*Fonsecaea pedrosoi*		
		Saprophytic form	Muriform cells in vivo	Muriform cells in vitro
1	Control	−	−	−
2	Alkaline phosphatase	＋	−	−
3	Esterase (C4)	＋	±	±
4	Esterase lipase (C8)	−	−	−
5	Lipase C14	−	−	−
6	Leucine arylamidase	±	−	−
7	Valine arylamidase	−	−	−
8	Cystine arylamidase	−	±	±
9	Trypsin	−	−	−
10	α-Chymotrypsin	−	−	−
11	Acid phosphatase	＋	＋	＋
12	Naphthol-AS-BI-phosphohydrolase	＋	＋	＋
13	α-Galactosidase	−	−	−
14	β-Galactosidase	−	−	−
15	β-Glucuronidase	−	−	−
16	α-Glucosidase	−	＋	＋
17	β-Glucosidase	＋	＋	＋
18	N-acetyl-β-glucosaminidase	−	−	−
19	α-Mannosidase	＋	−	−
20	α-Fucosidase	−	−	−

* According to the API ZYM protocol, the reading criteria for the positive or negative results were mentioned here. Negative cupule: colorless or pale yellow; positive cupule: violet for numbers 2–5, 11, 13, 14, 16, 17, 19, and 20; orange for 6–10; blue for 12 and 15; and brown for 18.

### Statistical methods.

For the analysis of footpad swelling and fungal loads in the footpad, data were represented as the mean ± standard error of the mean. Statistical comparisons were performed using univariate analysis of variance (ANOVA) for compatibility group design and least significant difference (LSD) *t*-test. A *P*-value < 0.05 was considered as significant, and *P*-value < 0.01 as highly significant. Statistical graphs were drawn by GraphPad Prism software in the present study. Furthermore, Power Analysis software was used to determine the sample size, and *n* > 3 in each group was enough (α = 0.05; 1−β = 0.9).

## RESULTS

### In vivo–transformed muriform cells can propagate by dividing and cause further damage to the originally infected footpads of athymic (nu/nu−) BALB/c mice.

**Figure 1.**
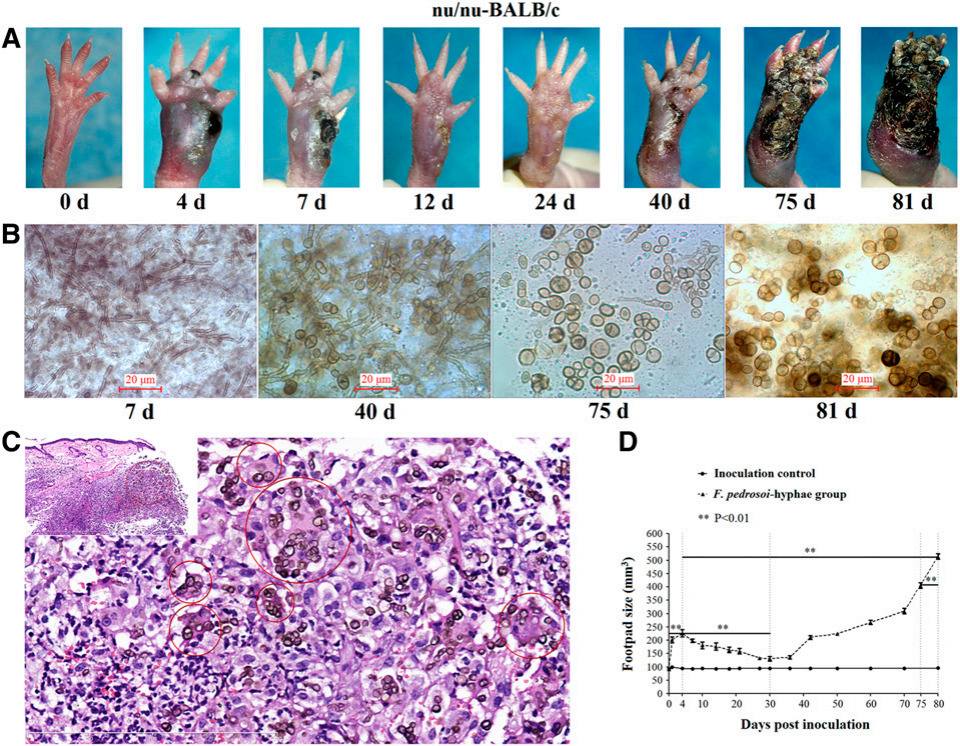
Muriform cells of *Fonsecaea pedrosoi* can cause invasive damage to the footpads of nu/nu-BALB/c mice without reverse transformation into hyphal form. (**A**) 100 μL of 1.5 × 10^8^ colony-forming units/mL *F. pedrosoi* hyphae was subcutaneously inoculated in the footpad of athymic (nu/nu) BALB/c mice (*n* = 5). Pre- (0 day) and postinoculation images for the same individual were taken at the indicated days. The BALB/c mice inoculated with 100 μL normal saline were set as inoculation control (*n* = 5). (**B**) Morphological analysis of this causative agent in the purulent secretion obtained from the same infected footpad as described in (**A**) at the indicated days. Scale bar = 20 μm. (**C**) Tissue section and HE staining (×400) of the same infected footpad as described in (**A**) and (**B**) at 81 days postinoculation. The causative agents phagocytized by the histiocyte-like cells were indicated by red circles. (**D**) Graph showing the footpad size measured with caliper following infection with *F. pedrosoi* hyphae for an 81-day observation period. Data represent the mean ± standard error of the mean (SEM) (*n* = 5 at each indicated time point), and statistical analysis was performed using univariate ANOVA for compatibility group design and LSD *t*-test. Highly significant: ** This figure appears in color at www.ajtmh.org.

**Figure 2.**
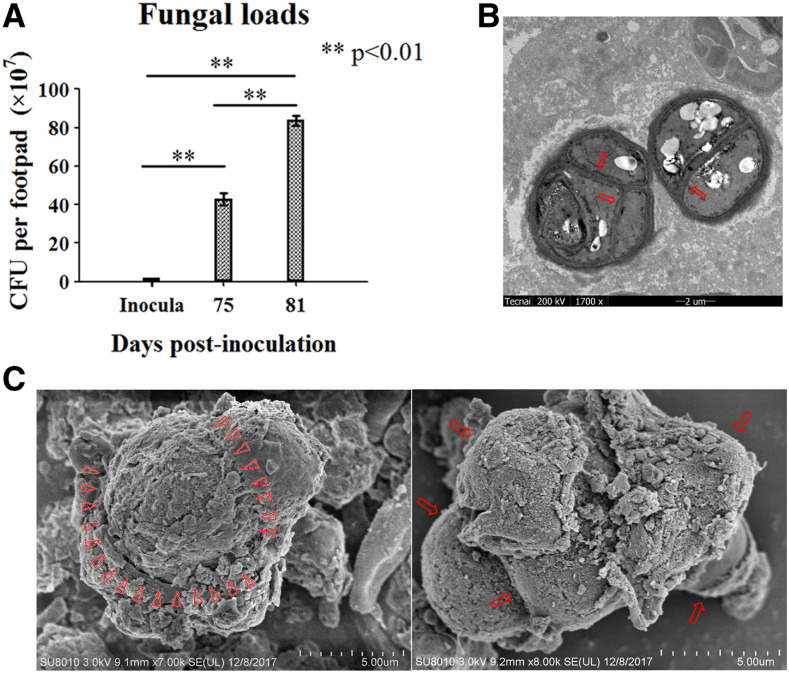
Fungal loads increased significantly with the maintenance of muriform cells in the infected footpads. (**A**) Fungal loads in the infected footpad tissues were detected, respectively, at 75 and 81 days postinoculation and represented as colony-forming unit. Data represent the mean ± SEM (*n* = 4 at each indicated time point), and statistical analysis was performed using univariate ANOVA and LSD *t*-test. Highly significant: ** *P* < 0.01. (**B** and **C**) Transmitting electronic microscope (TEM) and SEM examinations for muriform cells obtained from the infected footpad tissue at 81 days postinoculation. (**B**) TEM examination showed typical thick-walled muriform cells with cross- or single septation, as indicated by red arrows. (**C**) SEM examination showed the division line that delimits the portion of cell wall involved in septation process, as indicated by red triangles (**C**, left panel), and the daughter cells reproduced by dividing were indicated by red arrows (**C**, right panel). This figure appears in color at www.ajtmh.org.

For the athymic (nu/nu−) BALB/c mice subcutaneously infected with *F. pedrosoi* hyphae, swollen footpads occurred and developed with ulcers and necrosis from day 0 to day 4 postinoculation, and then footpad swelling decreased steadily with clinical improvement till day 30 postinoculation (Figure 1A and D). However, worsening of footpad lesions was observed with significantly increased footpad swelling during the next part of the whole 80-day observation period (Figure 1A and D). Fungal examination of pus fluid obtained from the same infected footpad at the indicated time points showed that the swelling chlamydospores with cross-septation occurred at terminal and middle positions of hyphae at 40 days postinoculation, and transformation of this agent into muriform cells with multi-septations was consecutively observed at 75 and 81 days postinoculation in contrast with the typical hyphal form of *F. pedrosoi* at 7 days postinoculation (Figure 1B). As compared with the infected footpad at 75 days postinoculation, clinical observation and histological sections showed that the tiptoes of the infected footpad were further invaded by the agents which presented themselves as muriform cells in tissue at 81 days postinoculation (Figure 1B and C). Meanwhile, the fungal load in the infected footpad increased significantly at 81 days postinoculation when compared with that at 75 days postinoculation (Figure 2A). Transmitting electronic microscope image revealed the well-preserved, thick-walled muriform cells with single or cross-septation surrounded by mouse tissue components, and the inner layer of fungal cell wall was involved in the formation of septation that is peculiar of planate division, as shown by the red arrow (Figure 2B). SEM examination for the muriform cells obtained in vivo also showed the division line delimitating the cell wall portion involved in septation process, as indicated by red triangles (Figure 2C, left panel). In addition, it was observed that the muriform cells can reproduce daughter cells by dividing, as shown by red arrows (Figure 2C, right panel). The fungi recovered from the infected footpad tissues have 100% sequence identity at “ITS1+5.8S rRNA+ITS2” region with the *F. pedrosoi* WH10-002 (GenBank number: GQ420654.1).

### In vitro–induced muriform cells of *F. pedrosoi* can divide to form daughter cells, and, meanwhile, the daughter cells can produce buds and change into elongated hyphae reversely.

**Figure 3.**
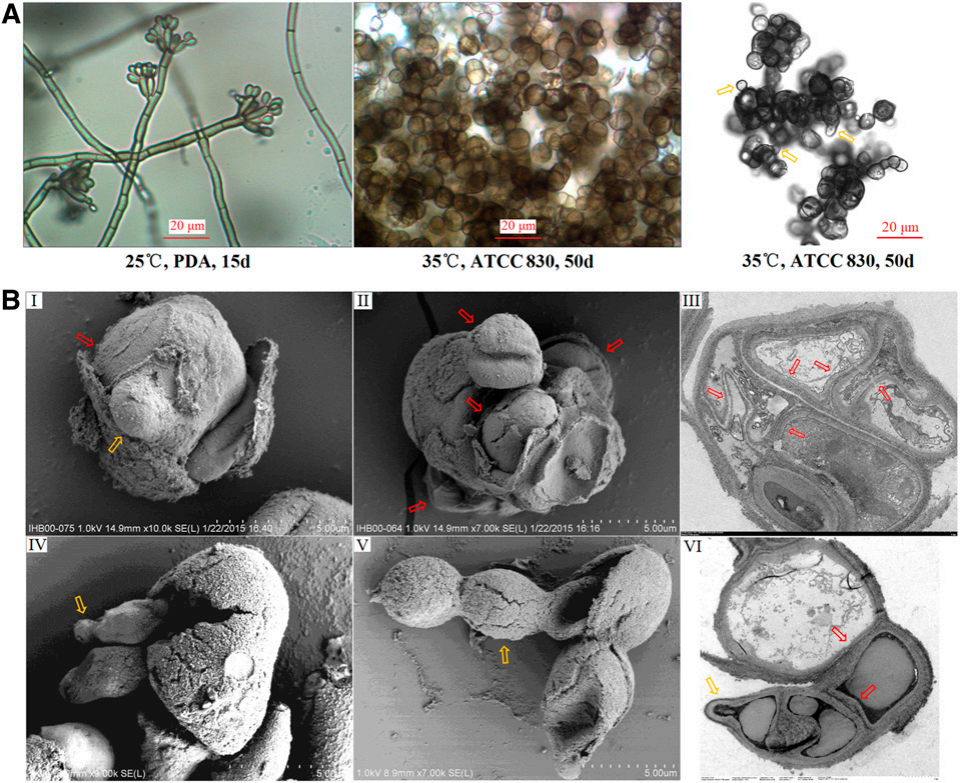
In vitro–induced muriform cells of *Fonsecaea pedrosoi* were able to propagate by dividing, and, meanwhile, the daughter cells had the potential to produce buds and change reversely into the elongated hyphae. (**A**) Normal or confocal optical microscope was used to characterize the morphology of saprophytic *F. pedrosoi* growing on potato dextrose agar (left panel) and in vitro–induced muriform cells in ATCC 830 medium (middle and right panels) (×400), scale bar = 20 μm. The budding produced by muriform cells was indicated by the yellow arrows (right panel). SEM examination showed the muriform cells can reproduce daughter cells by dividing (**B**-**I**, **II**), as indicated by the red arrows, and, meanwhile, the daughter cells had the potential to produce buds and change reversely into elongated hyphae (**B**-**I**, **IV**, **V**), as indicated by the yellow arrows. Transmitting electronic microscope examination showed the muriform cells with multi-septations, and the inner part of the thick-layered cell wall was involved in the formation of cellular septation (**B**-**III**, **VI**), as indicated by the red arrows. Simultaneously, the inner cell formed by septation had the potential to produce bud (**B**-**VI**), as indicated by the yellow arrow. Scale bar = 500 nm (**B**-**I**, **II**, **IV**, **V**) or 200 nm (**B**-**III**, **VI**). This figure appears in color at www.ajtmh.org.

The slide culture of *F. pedrosoi* WH10-002 growing on PDA at 25°C for 15 days showed characteristic dematiaceous hyphae originating terminal cylindrical conidiophores with small subhyaline conidia which were produced by acropetal budding and sympodially arranged on short denticles (Figure 3A, left panel).

By contrast, morphological transformation of this agent from its hyphal form into brownish, multi-septated muriform cells was observed in ATCC 830 medium plus 50 μg/mL Nikkomycin Z at 35°C within 50 days postinoculation (Figure 3A, middle panel). And, meanwhile, the buds produced by muriform cells can be also observed, as indicated by the yellow arrows (Figure 3A, right panel). SEM further showed that the muriform cells can reproduce more than one daughter cell by dividing, as indicated by the red arrows (Figure 3B-I, II), and the daughter cells have the potential to produce buds and change into elongated hyphae reversely, as indicated by the yellow arrows (Figure 3B-I, IV, V). What is more, TEM examination showed that the inner part of the thick-layered cell wall was involved in the formation of cellular septation, as was similar to that of muriform cells in vivo, and indicated by the red arrows (Figure 3B-III, VI). Simultaneously, it was observed that the inner cell formed by cellular septation can produce buds reversely, as was indicated by the yellow arrow (Figure 3B-VI), and coincided with the budding phenomenon observed by SEM.

### *Fonsecaea pedrosoi* muriform cells as well as the saprophytic hyphae have metabolic activities.

**Figure 4.**
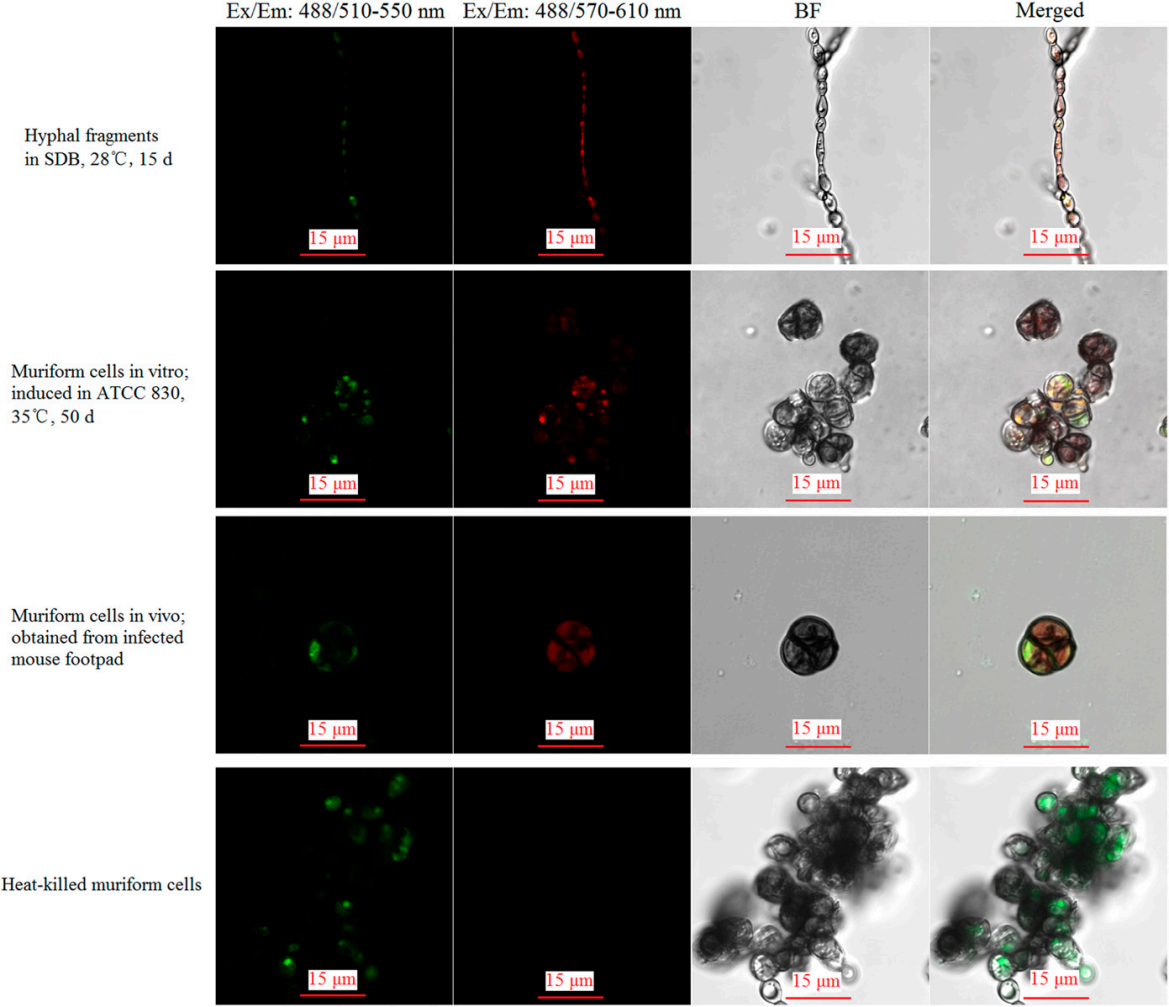
Muriform cells obtained in vivo and in vitro as well as the saprophytic hyphae have metabolic activities. The metabolic activities of muriform cells obtained in vivo and in vitro as well as saprophytic hyphae were detected by FUN-1 stain using a confocal microscope. According to the protocol, the excitation length was set as 488 nm; the original green fluorescence emission was observed at 510–550 nm range, whereas the red-shift fluorescence, which can reflect the cellular metabolic activities according to the protocol, was observed at 570–610 nm range. BF = bright field; merged = merged figure. Scale bar = 15 μm; magnification = 1,000. This figure appears in color at www.ajtmh.org.

The intracellular distribution of red fluorescence and green fluorescence was observed both in the muriform cells obtained in vivo and in vitro as well as live hyphal fragments (Figure 4). For the heat-killed *F. pedrosoi* hyphae, intracellular distribution of red fluorescence cannot be detected, although the original green fluorescence emission by FUN-1 dye can be observed (Figure 4). According to the protocol, the red shift of FUN-1 fluorescence reflects that the muriform cells obtained in vivo and vitro as well as the saprophytic hyphae have metabolic activities.

### The exoenzyme profiles constituted by muriform cells in vivo and in vitro were quite different from that constituted by hyphae.

**Figure 5.**
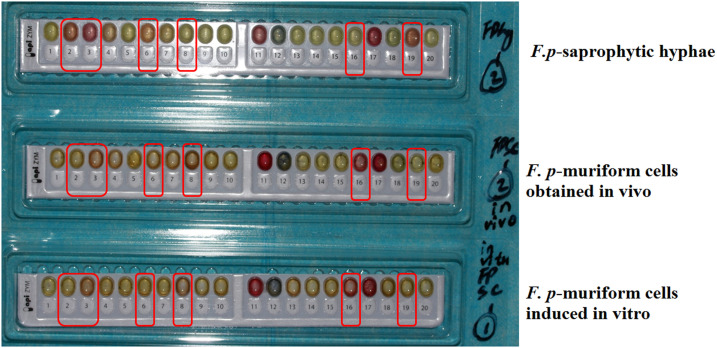
The exoenzyme profiles of muriform cells obtained in vivo and in vitro were different from that of saprophytic hyphae. The exoenzyme profiles of muriform cells obtained in vivo and in vitro as well as saprophytic hyphae were detected by API ZYM strip. Briefly, the suspensions of *Fonsecaea pedrosoi* hyphae and muriform cells adjusted to 2.0 × 10^8^ colony-forming units/mL in 2 mL of API suspension medium (Ref 70700, BioMericux, France) and dispensed into each cupule of the strip. After incubation for 5 hours at 37°C, ZYM A (Ref 70494, BioMericux, France) and ZYM B (Ref 70493, BioMericux, France) reagents were, respectively, added into each cupule according to the protocol. The reading criteria for the positive and negative results were as follows. Negative cupule: colorless or pale yellow; positive cupule: violet for numbers 2–5, 11, 13, 14, 16, 17, 19, and 20; orange for 6–10; blue for 12 and 15; and brown for 18. This figure appears in color at www.ajtmh.org.

The 19 exoenzyme items detected by API ZYM strip are listed in Table 1. The profiles of these exoenzymes produced by muriform cells in vivo and in vitro were basically similar, which was quite different from that constituted by hyphae (Table 1, Figure 5). Briefly, different enzymatic activities of alkaline phosphatase, esterase (C4), leucine arylamidase, cystine arylamidase, α-glucosidase, and α-mannosidase were observed in muriform cells and saprophytic hyphae (Table 1, Figure 5).

### The muriform cells in the mouse footpad transformed reversely into the hyphal form after intraperitoneal administration of CTX.

**Figure 6.**
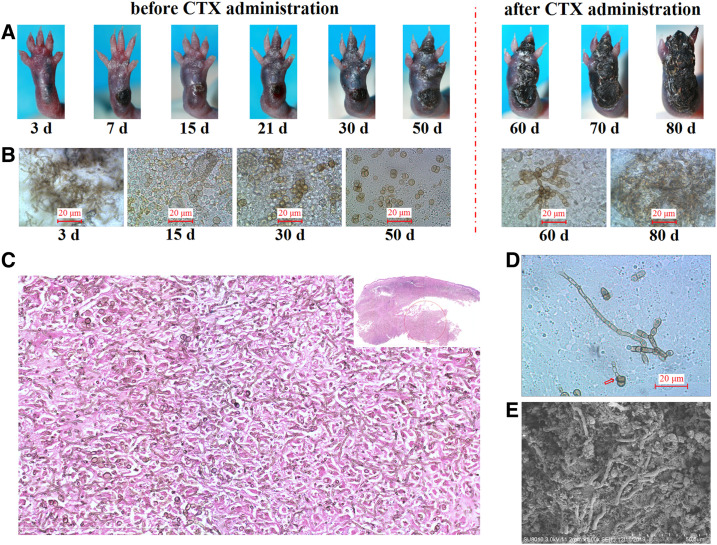
Intraperitoneal administration of cyclophosphamide (CTX) linked to the morphological change of muriform cells into hyphal form in the footpad of nu/nu-BALB/c mice. (**A**) Each mouse (*n* = 5) was subcutaneously inoculated with 100 μL of *F. pedrosoi* hyphal fragments (1.5 × 10^8^ colony-forming units/mL) and, then, intraperitoneally administered with 300 μL of CTX (50 mg/kg) every other day from 50 till 80 days postinoculation after transformation of hyphal inocula into muriform cells at 50 days postinoculation. The images of lesion development for the same mouse footpad were taken at indicated time points. (**B**) Morphological analysis of the agent in the purulent secretion obtained from the same infected footpad as described in **(A)** at the indicated days. Scale bar = 20 μm. **(C)** Histological examination for the infected footpad was taken at 80 days postinoculation, and HE staining showed that most agents presented as elongated hyphae and disseminated throughout the footpad tissue without obvious infiltration of inflammatory cells. (**D**) Hyphal extending from the muriform cell was observed in the purulent fluid at 80 days postinoculation after CTX administration, as indicated by the red arrow. (**E**) SEM examination for the infected footpad at 80 days postinoculation also showed that the agents penetrated throughout the footpad tissue as the hyphal form. This figure appears in color at www.ajtmh.org.

For the athymic (nu/nu−) BALB/c mice subcutaneously inoculated with *F. pedrosoi* hyphae and then administered with CTX repeatedly from 50 to 80 days postinoculation, swollen footpads occurred and developed with ulcers and necrosis from day 0 to day 50 postinoculation (Figure 6A). After CTX administration, worsening of the footpad lesion can be observed with the gangrene and breakdown of tiptoes from 50 to 80 days postinoculation (Figure 6A). Fungal examination of pus fluid obtained from the same infected footpad at the indicated time points showed that the hyphal inocula had gradually transformed into the swelling chlamydospores with single septation or muriform cells from 3 to 50 days postinoculation (Figure 6B). By contrast, with repeated intraperitoneal administration of CTX from 50 to 80 days postinoculation, the agents in the pus gradually changed reversely into the toruloid cells or septated hyphae (Figure 6B). At the time of mouse sacrifice (80 days postinoculation), biopsy examination and HE staining further showed that most agents in the same infected footpad as mentioned earlier presented themselves as elongated hyphae and disseminated throughout the footpad tissue without obvious infiltration of inflammatory cells (Figure 6C). Simultaneously, hyphal extension from the muriform cell was clearly observed in the pus, as indicated by the red arrow (Figure 6D). Furthermore, SEM examination for the footpad also showed that the agents presented as hyphal form and penetrated throughout the tissue (Figure 6E).

## DISCUSSION

Chromoblastomycosis is considered as an implantation mycosis, and the patients usually acquire the infection through an injury from plant material.^3,25,26^ Of note, some previous study showed that the inoculated *F. pedrosoi* can differentiate into muriform cells in the epidermis of *Mimosa pudica* plant, from which the agent was isolated.^25–27^ In the present study, we further demonstrated that *F. pedrosoi* can reproduce daughter cells as muriform cells in mouse tissue—and cause further damage to the footpad without reverse transformation into hyphal form. All these data might provide some clues for investigating whether the muriform cells can penetrate the skin through the plant vehicle and initiate the disease.

When compared with saprophytic mycelia or conidia, the muriform cells had special characteristics including optimized surface/volume ratio favoring increased melanin deposition, and higher acid phosphatase activity,^3,10,28^ which contributed to their immune escape and the chronicity of this disease. Notably, low level of Interferon (IFN)-γ and inefficient T-cell proliferation were observed in patients with a severe form of chromoblastomycosis, albeit that the agents usually invaded in laborers with fully functional immunity through traumatic skin lesions.^3,29,30^ Our previous study further demonstrated that the exclusive accumulation of chitin on the outer cell wall of muriform cells in vitro and in vivo was involved in an inhibited IFN-γ production and, thus, the recalcitrance of experimental mouse chromoblastomycosis.^11^ Therefore, we hypothesized that the self-reproducing ability of muriform cells by dividing may be linked to the chronic development of chromoblastomycosis.

Furthermore, the present study showed that muriform cells obtained in vitro and in vivo can reproduce daughter cells by dividing and have different exoenzyme profiles from that of saprophytic hyphae. Accordingly, we inferred that the muriform cells cannot be considered merely as compacted masses of latent hyphae, which was derived from the term sclerotic cells or sclerotia.^3^ And it should be pointed out that although obvious acid phosphatase activity in muriform cells in vivo and in vitro was detected by API ZYM strip Ref 25200, BioMericux, France, and was in agreement with the previous studies,^28^ the alkaline phosphatase activity was mainly detected in hyphal fragments rather than the muriform cells. We inferred that this might be associated with the microenvironmental pH values in vitro and in vivo, and at least the pH value (pH = 5.5) in the ATCC 830 medium was suitable for the activation of acid phosphatase but not alkaline phosphatase. In addition, considering the common characteristics mentioned earlier between in vivo and in vitro muriform cells, it is possible that the in vitro–induced muriform cell might be taken as a tool to analyze the pathogenesis of chromoblastomycosis.

Although the presentation of *F. pedrosoi* as hyphal form in tissue can be observed in some immune-privileged organs or immunosuppressed patients,^15–17,31,32^ it still leaves much to be determined whether there exists a linkage between the parasitic form of chromoblastomycosis agents and host immune status till now. Interestingly, some studies suggested that the reexposure of C57BL/6 mice to hyphae/conidida of *F. pedrosoi* developed lesions with muriform cells and caused a delayed resolution of the infection with relatively lower degree of neutrophilic process in comparison to mice that were not reexposed.^33^ In the present study, our data also showed that the hyphal inocula of *F. pedrosoi* gradually transformed into the muriform cells in the footpad tissue of athymic mouse, where the macrophages were recruited obviously and phagocytized the agents. By contrast, after repeated intraperitoneal administration of CTX, an immunosuppressant widely used in experimental mouse,^34,35^ most *F. pedrosoi* muriform cells change into the elongated hyphae reversely and disseminated throughout the footpad tissue without any infiltration of inflammatory cells. Accordingly, although there remain great differences in the species distribution and the ability to transform into muriform cells between chromoblastomycosis and phaeohyphomycosis agents,^3,36,37^ we infer that the host immune status and the pattern of inflammatory cells in tissue might also influence the parasitic morphology of specific chromoblastomycosis agent including *F. pedrosoi*.

In sum, the present study provides novel evidence for the biological activities and reproduction characteristics of *F. pedrosoi* muriform cells in vivo and in vitro, and, therefore, extends our understanding of the refractoriness of chromoblastomycosis. Furthermore, it should be interesting and necessary to compare the difference in invasion-related factors between the saprophytic form and parasitic muriform cells of chromoblastomycosis agents at transcriptome and proteome levels in the next work.
